# Asymptomatic Presacral Paraganglioma: Management of an Unpredictable Intraoperative Finding

**DOI:** 10.1055/s-0040-1712545

**Published:** 2020-06-30

**Authors:** Athina A. Samara, Alexandros Diamantis, Dimitrios Symeonidis, Athanasios Anagnostou, Andreas Marios Diamantis, Georgios Mavrovounis, Konstantinos Tepetes

**Affiliations:** 1 Department of Surgery, University Hospital of Larissa, Larissa, Greece

**Keywords:** paraganglioma, secreting tumor, presacral paragangliomas, 131-I MIBG, neoplasm

## Abstract

Paragangliomas are rare neuroendocrine tumors originating from the embryological neural crest. We report a rare case of a patient with an asymptomatic presacral mass (incidentaloma) who experienced an unpredictable intraoperative hypertensive crisis after manipulation of the tumor. Presacral neoplasms pose a diagnostic and therapeutic challenge due to their obscure anatomical location and the difficulty in performing an R0 excision. Furthermore, the management of asymptomatic paragangliomas requires a high level of clinical suspicion and expertise due to potential life-threatening intraoperative complications.

Paragangliomas are rare neuroendocrine neoplasms arising from extra-adrenal neural crest-derived cells of the sympathetic and parasympathetic nervous system. When paragangliomas are located in the adrenal medulla, they are termed as pheochromocytomas. 1 Their incidence is estimated to be approximately two to eight cases per million 1 with most cases considered sporadic, thus, they can be part of a familial syndrome such as neurofibromatosis, MEN 2 (multiple endocrine neoplasia 2), Carney–Stratakis dyad, von Hippel–Lindau disease, etc. 2 Paragangliomas occur most commonly in the head and neck region, but can be found anywhere along the sympathetic chain. 3 Symptoms of catecholamine secretions such as hypertension, hyperhidrosis, and hyperglycemia can be present. 4 While only 15% of the patients remain asymptomatic, patients are typically diagnosed during imaging conducted for other medical purposes. 3 Presacral paragangliomas are rarer still and pose a diagnostic and therapeutic challenge due to their obscure anatomical location and the difficulty of performing an R0 resection. 5

We present a rare case report of an asymptomatic presacral mass causing an unpredictable acute hypertensive crisis intraoperatively, which is unmanageable with medical treatment.

## Case Report

An asymptomatic 65-year-old Caucasian male presented with a pelvic mass which had been diagnosed during a routine lower abdomen ultrasonography due to benign prostatic hypertrophy. A digital rectal examination revealed a mass displacing the posterior rectum wall, without involving the rectal mucosa. Upon physical examination there were no signs of “Café au lait spots,” other signs of neurofibromatosis, or other familial syndromes associated with neuroendocrine tumors. The patient's past medical history revealed both arterial hypertension and benign prostatic hyperplasia.

**Fig. 1:**
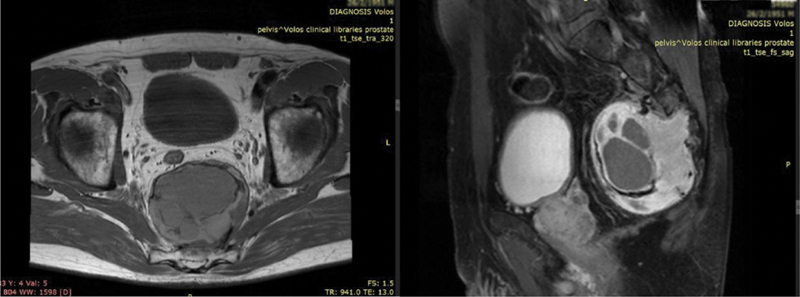
Lower abdomen magnetic resonance imaging (MRI). A well-circumscribed heterogeneous signal intensity mass arising from the intraforaminal segment of the S3 nerve root, measuring 7.5 × 6.4 × 7.7 cm.

Lower abdomen magnetic resonance imaging (MRI) revealed a well-circumscribed heterogeneous signal intensity mass arising from the intraforaminal segment of the S3 nerve root, measuring 7.5 × 6.4 × 7.7 cm ( Fig. 1 ). Displacement of the rectum was observed with no signs of invasion. According to the radiologist, these findings indicated a possible malignant peripheral nerve sheath tumor.

The patient underwent an open anterior surgical approach. Unexpectedly, after entering the presacral space and manipulating the identified lesion, acute hypertensive crisis (systolic blood pressure: 300 mm Hg) and tachycardia with documented pulses >210 occurred. Due to failure in controlling and normalizing the patient's tension and heart rhythm, the operation was terminated without excising the mass. Postoperatively, all necessary exams were conducted to identify a catecholamine-secreting tumor. Over a 24 hour-period urine catecholamines were measured at 1,313 mg (three times above the normal rate), adrenaline at 257 μg (10 times above the normal rate), noradrenaline at 130 μg, and dopamine at 918 μg (two times above the normal rate). CT scans of the head, neck, chest, and abdomen were performed to exclude the possibility of neural crest tumors in other sites. This was followed by the initiation of treatment with α-blockers (phenoxybenzamine) and β-blockers (propranolol). After 5 days the patient was discharged and given instructions to continue his treatment with both α and β-blockers.

One month later a second laparotomy was performed. The lesion was fully excised but due to profuse, uncontrollable bleeding of the presacral venous plexus the abdomen was packed, and the patient was sent to the intensive care unit (ICU). After a 24-hour period, the unpacking procedure was performed and was uneventful, and the patient was transferred to the Surgery Department. Following a full recovery, the patient was discharged on the eighth postoperative day.

Upon histological examination, characteristics of a paraganglioma with the typical zellballen cells were identified. Nuclear atypia and pleomorphism were present; mitotic activity was high (10/10 high power fields X 40 Zeiss) and vascular invasion was also observed. Immunohistochemically, the tumor was SMA (−), HMB45 (−), AE1 (−), AE3 (−), CKIT (−), MELAN A (−), DESMIN (−), CD68 (−), EMA (−), VIMENTIN (+), and S100 (+). Tumor analysis of succinate dehydrogenase (SDH) subunits was not available in the Pathology Laboratory of our hospital.

**Fig. 2:**
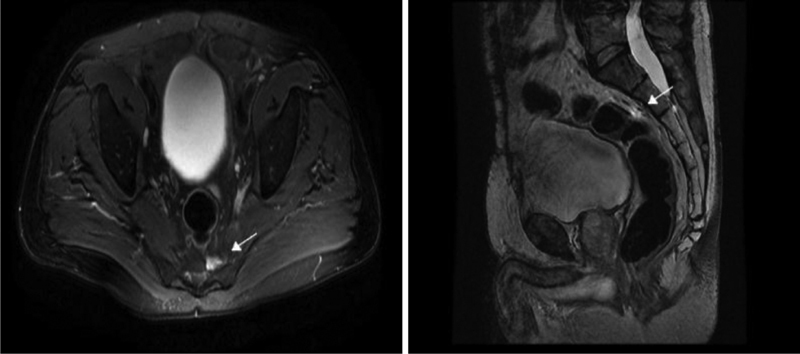
MRI (after 3 months): High contrast material uptake approximately 1 cm diameter in the anatomical position of the excised lesion ( *white arrow* ). MRI, magnetic resonance imaging.

**Fig. 3:**
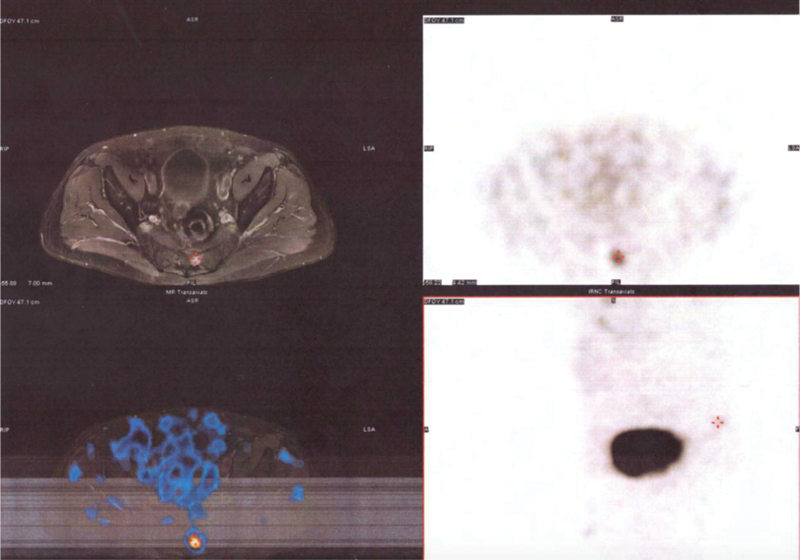
SPECT-CT (Single-photon emission computed tomography) with 99mTc-Tektrotyd: small intake in the area of the resection ( *red cross* ).

Three months later, the patient's urine catecholamines were normalized. Thus, the following MRI revealed signs of high contrast material uptake with a 1-cm diameter, in the anatomical position of the previously excised lesion ( Fig. 2 ). SPECT-CT (single-photon emission computed tomography [CT]) with 99mTc-Tektrotyd had shown a small intake in the area of the resection ( Fig. 3 ). Three cycles of ^131^ I-metaiodobenzylguanidine ( ^131^ I-MIBG) treatment followed, and in a SPECT-CT conducted 1 month later, there was no radiodrug intake. The patient has since been closely followed-up, undergoing MRI and urine catecholamine tests twice per year; 4 years later the patient remains disease-free.

In accordance with the existing guidelines on pheochromocytoma and paraganglioma management, genetic testing for SDH was recommended to the patient. Taking into consideration that the patient had no children or other first-degree relatives, and the high cost of the test which the patient was unable to afford, he refused genetic testing. A shared decision was reached to proceed with close biochemistry and radiological follow-up of the patient on a biannual basis.

## Discussion

Pheochromocytomas and paragangliomas (PPGLs) are neuroendocrine tumors arising from adrenomedullary chromaffin cells and extra-adrenal chromaffin cells respectively, which produce catecholamines. Approximately 80 to 85% of chromaffin-cell tumors are pheochromocytomas, whereas only 15 to 20% are paragangliomas. The exact prevalence, however, remains unclear, with 0.05 to 0.1% detected in autopsy studies, which went undetected during the individual's life. 6 While up to 40% of the patients possess a familial mutation in a known susceptibility gene—which is more than any other solid tumor—most cases are considered to be sporadic. 7

Compared with pheochromocytomas, only 1% of paragangliomas are functional and produce catecholamines, with the majority being asymptomatic or having mass effects such as vague abdominal pain. 2 In patients with a paraganglioma located below the neck level, the classic signs and symptoms associated with catecholamine excess include headache (26%), palpitations (21%), sweating (25%), and episodic hypertension (64%); however, only one-third of patients will suffer from these symptoms. Other less obvious symptoms associated with catecholamine excess include hyperglycemia, panic attacks, fever, weight loss, myocardial infarctions, osteolytic bone metastases, and Raynaud's phenomenon. 8

At least 10% of pheochromocytomas and sympathetic paragangliomas are malignant, although rates of malignancy differ according to the patient's hereditary background. Lymph nodes, skeleton, liver, and lungs are the most frequent sites of metastases. 9 CT with contrast provides an excellent initial method for the localization of paragangliomas (sensitivity 88–100%) and an MRI can be useful when a CT is contraindicated. 6 The use of ^123^ I-MIBG 1 scintigraphy is recommended as a functional imaging modality in patients with either metastatic PPGLs or an increased risk for metastatic disease, multifocal, and recurrent disease. 6 In addition, a 18F-FDG PET/CT (18F-fluorodeoxyglucose positron emission tomography/computed tomography) scan is recommended in patients with metastatic disease. 6

Primary surgical excision remains the treatment of choice for resectable paragangliomas. 10 Therapeutic use of ^131^ I-MIBG could offer a safe and effective option for patients with unresectable or metastatic PPGLs. 11 12 13 Preoperative preparation is crucial to decrease the intraoperative hypertensive spikes, and minimize the perioperative adverse events and overall morbidity. 14

Interval surveillance of these patients is similar to every other patient with neuroendocrine tumors. Following an R0 excision, blood pressure and levels of catecholamines or chromogranin (in patients with normal catecholamines preoperatively) should be measured every 3 months during the first year, every 6 months until the third year, and then annually for up to 10 years. In addition, abdominal/pelvic CT or MRI scans with contrast, or FDG-PET/CT scans can be considered. These follow-up exams can be performed at an earlier stage if symptoms dictate it. Moreover, patients with hereditary paraganglioma may require more frequent follow-up. 10

In this report, in addition to presenting a rare case, we would like to focus on the surgeon's dilemma regarding the optimal surgical approach for the patient, which as the MRI dictates ( Fig. 1 ), would ideally be an en-bloc sacrectomy below the segment S2. However, this approach would lead to excess morbidity and deterioration of the patient's quality of life, due to the inevitable injury of the sacral nerve plexus. 15 16 Therefore, after discussing all possible alternatives with the patient, he was first treated through excision of the mass, and subsequently, the residual disease was treated with ^131^ I-MIBG therapy which showed a remarkable response.

## Conclusion

Presacral paragangliomas pose both a diagnostic and therapeutic challenge because of the obscure anatomical location and the difficulty in obtaining an R0 excision. Furthermore, a high level of clinical suspicion of a possible secreting tumor, as well as expertise in management is required due to potential life-threatening intraoperative complications.

## References

[1] Delellis R A Lloyd R V Heitz P U World Health Organization Classification of Tumours. Pathology and Genetics of Tumours of Endocrine Organs Lyon IARC Press 2004

[2] Mantas D Kandilis A Charalampoudis P Nonfunctioning symptomatic paraganglioma: is there an optimal follow-up for patients with extra-adrenal benign paragangliomas J Surg Case Rep 2014 2014 09 rju092 25194052 10.1093/jscr/rju092 PMC4155394

[3] Pedroso C Robalo R Sereno P Barros C Marques C A rare abdomino-pelvic tumor: paraganglioma Acta Med Port 2015 28 01 114 116 25817505 10.20344/amp.5403

[4] Guo Q Li B Guan J Yang H Wu Y Intraoperative diagnosis of functional retroperitoneal multiple paraganglioma: a case report Oncol Lett 2012 4 04 829 831 23205108 10.3892/ol.2012.795 PMC3506663

[5] Saxena D Pandey A Bugalia R P Management of presacral tumors: our experience with posterior approach Int J Surg Case Rep 2015 12 37 40 25996775 10.1016/j.ijscr.2015.05.015 PMC4486097

[6] Lenders J W Duh Q Y Eisenhofer G Pheochromocytoma and paraganglioma: an endocrine society clinical practice guideline J Clin Endocrinol Metab 2014 99 06 1915 1942 24893135 10.1210/jc.2014-1498

[7] Fishbein L Pheochromocytoma and paraganglioma: genetics, diagnosis, and treatment Hematol Oncol Clin North Am 2016 30 01 135 150 26614373 10.1016/j.hoc.2015.09.006

[8] Lee J A Duh Q Y Sporadic paraganglioma World J Surg 2008 32 05 683 687 18224469 10.1007/s00268-007-9360-4

[9] Plouin P F Amar L Dekkers O M European Society of Endocrinology Clinical Practice Guideline for long-term follow-up of patients operated on for a phaeochromocytoma or a paraganglioma Eur J Endocrinol 2016 174 05 G1 G10 27048283 10.1530/EJE-16-0033

[10] NCCN Clinical Practice Guidelines in Oncology (NCCN Guidelines). J Natl Compr Canc Netw 2018;16(06):693–702

[11] Carrasquillo J A Pandit-Taskar N Chen C C I-131 metaiodobenzyl guanidine therapy of pheochromocytoma and paraganglioma Semin Nucl Med 2016 46 03 203 214 27067501 10.1053/j.semnuclmed.2016.01.011

[12] van Hulsteijn L T Niemeijer N D Dekkers O M Corssmit E P (131)I-MIBG therapy for malignant paraganglioma and phaeochromocytoma: systematic review and meta-analysis Clin Endocrinol (Oxf) 2014 80 04 487 501 24118038 10.1111/cen.12341

[13] Jimenez C Erwin W Chasen B Targeted radionuclide therapy for patients with metastatic pheochromocytoma and paraganglioma: from low-specific-activity to high-specific-activity Iodine-131 metaiodobenzylguanidine Cancers (Basel) 2019 11 07 E1018 31330766 10.3390/cancers11071018 PMC6678905

[14] Ramachandran R Rewari V Factors affecting the haemodynamic behaviour of patients undergoing pheochromocytoma and paraganglioma removal: a review Cardiovasc Endocrinol 2017 6 02 73 80 31646123 10.1097/XCE.0000000000000090 PMC6768518

[15] Dziki Ł Włodarczyk M Sobolewska-Włodarczyk A Presacral tumors: diagnosis and treatment—a challenge for a surgeon Arch Med Sci 2019 15 03 722 729 31110540 10.5114/aoms.2016.61441 PMC6524179

[16] Sasikumar A Bhan C Jenkins J T Antoniou A Murphy J Systematic review of pelvic exenteration with en bloc sacrectomy for recurrent rectal adenocarcinoma: R0 resection predicts disease-free survival Dis Colon Rectum 2017 60 03 346 352 28177998 10.1097/DCR.0000000000000737

